# Sparsity as Cellular Objective to Infer Directed Metabolic Networks from Steady-State Metabolome Data: A Theoretical Analysis

**DOI:** 10.1371/journal.pone.0084505

**Published:** 2013-12-31

**Authors:** Melik Öksüz, Hasan Sadıkoğlu, Tunahan Çakır

**Affiliations:** 1 Department of Bioengineering, Gebze Institute of Technology, Gebze, Kocaeli, Turkey; 2 Department of Chemical Engineering, Gebze Institute of Technology, Gebze, Kocaeli, Turkey; Leibniz-Institute for Farm Animal Biology (FBN), Germany

## Abstract

Since metabolome data are derived from the underlying metabolic network, reverse engineering of such data to recover the network topology is of wide interest. Lyapunov equation puts a constraint to the link between data and network by coupling the covariance of data with the strength of interactions (Jacobian matrix). This equation, when expressed as a linear set of equations at steady state, constitutes a basis to infer the network structure given the covariance matrix of data. The sparse structure of metabolic networks points to reactions which are active based on minimal enzyme production, hinting at sparsity as a cellular objective. Therefore, for a given covariance matrix, we solved Lyapunov equation to calculate Jacobian matrix by a simultaneous use of minimization of Euclidean norm of residuals and maximization of sparsity (the number of zeros in Jacobian matrix) as objective functions to infer directed small-scale networks from three kingdoms of life (bacteria, fungi, mammalian). The inference performance of the approach was found to be promising, with zero False Positive Rate, and almost one True positive Rate. The effect of missing data on results was additionally analyzed, revealing superiority over similarity-based approaches which infer undirected networks. Our findings suggest that the covariance of metabolome data implies an underlying network with sparsest pattern. The theoretical analysis forms a framework for further investigation of sparsity-based inference of metabolic networks from real metabolome data.

## Introduction

While the majority of computational systems biology approaches use cellular networks as scaffolds to analyze omics data, some focus on investigation of the information content of omics data to recover the underlying biological network. These approaches, termed top-down systems biology [1], have been more widely applied to transcriptome data to infer gene-regulatory or signaling networks [2]–[5] whereas applications to metabolome data to discover metabolic networks are rather limited [6]–[8].

Network inference approaches can be grouped into two in terms of the directionality of the inferred network. A large group of approaches including similarity-based approaches such as partial Pearson correlation and mutual information infers undirected networks [9], [10]. Others use dynamic or multi-condition data with sophisticated/advanced experimental design [11], [12] to increase the information content of data, and hence attempt to infer directed cellular networks.

Steady-state data have also been a focus of reverse engineering approaches, but almost exclusively to infer undirected networks. Few examples use steady-state data only to infer partially directed networks [13]. Steady-state data, although leading to promising results, are generally considered to be less informative compared to dynamic or multi-conditional data. Therefore, the general trend is to employ other complicated experimental designs such as perturbation experiments [14], which may require higher costs.

Cellular networks have been shown to exhibit sparse structures [5]. This characteristics is also valid for condition-specific networks. Therefore, the sparsity information has already been used by some researchers in network inference approaches to further constrain solution space. For metabolic networks, a sparse structure means efficient use of cellular resources by minimizing the number of active reactions and, hence, the production of corresponding enzymes. The sparsity of metabolic networks has also been used to develop bottom-up modeling techniques to predict experimental data [15], [16].

In this work, we perform a theoretical study based on constraining observational steady state metabolome data with the sparsity information, and show the potential of such data to discover underlying metabolic networks with directionality information as well as the interaction strength of metabolite pairs. The results are demonstrated *in silico* for three different metabolic systems: brain glycolysis metabolism consisting of 12 metabolites, glycolytic pathway of *S. cerevisiae* with 13 metabolites, and central carbon metabolism of E. *coli* with 18 metabolites. The approach can also be used for the inference of other types of cellular networks (eg. based on transcriptome data), making it of wider interest for systems biology research.

## Methods

### Lyapunov Equation

A metabolic reaction network can be described by a set of nonlinear differential equations around its metabolites, **C**:
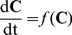
(1)


For systems around steady state, a linear approximation can be made to express the equation system in terms of Jacobian matrix, **J** [17] :
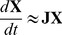
(2)with **X** = **C** – **C**_s_, and **C** shows concentrations fluctuating around steady state values, **C**_s_. Jacobian matrix holds very detailed information on the underlying network structure including (i) direction of interaction, (ii) nature of interaction (positive or negative), and (iii) strength of interaction. The (*i*, *j*)^th^ entry of a Jacobian matrix quantifies the influence of *j*th metabolite on the time behavior of metabolite *i*, hinting for interaction strength [6]:



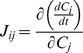
(3)
Eqn. (2) can also be expressed by a Langevin-type equation to explicitly account for small fluctuations [17].
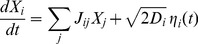
(4)where D_i_ shows the extent of fluctuations, and *η_i_* is a random number from unit normal distribution. Note that internal metabolites can show true, albeit small, natural fluctuations over time due to complex regulatory patterns in the cell [6], [17]–[19], or such fluctuations can be induced externally by eg. introducing small time-dependent fluctuations on temperature, pH or external glucose concentration of microbial growth systems.

As demonstrated by [20], Eqn. (4) can be written as follows at steady state, providing a link between the covariance matrix of metabolite data, **Г**, and Jacobian matrix:
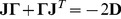
(5)


Eqn. (5), also known as Lyapunov Equation, is our basis for directed network inference based on steady state data since it puts a link between data-based covariance matrix and network structure, stored in Jacobian matrix. The equation was already demonstrated to be valid for metabolic networks as the covariance of data generated stochastically from a metabolic network was in agreement with the covariance calculated from this equation [17].

Eqn. (5) is a linear set of equations, and can further be arranged into a standard format of systems of equations:
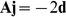
(6)


Here, **j** is the vectorized form of Jacobian matrix to be determined, with size of *n^2^*×*1*. Similarly, **d** is the vectorized form of the fluctuation matrix with *n^2^*×*1* in size. **A** is *n^2^*×*n^2^* matrix, including information on covariance values. Remember that matrix **A** is not in full rank since the covariance matrix has dependent entries. (See Supporting Information S1 for the derivation of Eqn. 6).

Eqn. (6) is underdetermined for solving the vectorized Jacobian matrix given covariance matrix since a covariance matrix is symmetric, and hence only has *n*(*n*+1)/2 independent entries while the vectorized Jacobian matrix has *n^2^* independent entries for an *n*-metabolite system. Namely, the degrees of freedom of the system is large and equal to *n*(*n*–1)/2. On the other hand, it was pointed out that if Jacobian matrix has number of zeros greater than the degrees of freedom of the system, than the system becomes overdetermined [21]. Since metabolic networks are sparse, this situation generally holds for metabolic networks. Our hypothesis is that, in addition to the minimization of the Euclidean norm of residuals, another proper objective function based on cellular network structure (eg. sparsity) can be simultaneously used to solve for the vectorized Jacobian matrix, and hence recover the underlying metabolic network.

### Obtaining Covariance Matrix

Lyapunov equation was already shown to hold for metabolic networks [17]. And, our goal is to be able to demonstrate that Jacobian matrix, and hence full network structure, can be recovered based on this equation via optimization, given the data-derived covariance matrix. In other words, we want to demonstrate the use of sparsity as an objective function by the cell. Therefore, we preferred to test this hypothesis via a theoretical analysis for a given exact covariance matrix. Since all the three metabolic systems analyzed have associated kinetic models, the corresponding true Jacobian matrices can easily be calculated. To this aim, we obtained the exact covariance matrix from Eqn. (5) for the true Jacobian matrix rather than deriving it from generated *in silico* data for the analyzed metabolic systems. Then, this covariance matrix and information on fluctuations (**d**) were used in our optimization framework to identify **j**. **d** was chosen as a vectorized matrix with diagonals being 0.005 in all simulations, implying small internal fluctuations of each metabolite at steady state.

Indeed, when we calculated Spearman correlation between the exact covariance matrix of yeast that we obtained from Eqn. (5) and the covariance matrix of *in silico* data generated for this system in another study [6], we obtained a value of 0.998; indicating very high overlap. However, *in silico* data generation using stochastic differential equations (SDE) of type Eqn. (4) requires the use of SDE solvers; and we observed that SDE solvers may not be stable for highly nonlinear kinetic models like the ones we work on. Small changes in fluctuation parameters lead to negative or imaginary concentration values for especially metabolites with low concentrations. That was one other reason why we preferred to use the exact covariance matrix which was obtained from Eqn. (5). Noise analysis on this covariance matrix was performed as discussed in Results and Discussion section in order to justify our choice on the use of exact covariance matrix.

### Constraining Solution Space using Correlations of Metabolite Pairs

The use of similarity-based network inference approaches (eg. correlation) to infer undirected metabolic networks from metabolome data showed that full-order partial Pearson correlation, also known as Graphical Gaussian Model (GGM), is the best performer among others studied [6]. A closer inspection of the results of that study showed that GGM-based similarity with a very stringent cut-off gives a perfect match with the corresponding network. i.e. Metabolite pairs with |R_GGM_| <0.001 do not have an edge in between in the real network, and pairs with |R_GGM_| >0.60 are linked in reality. We used this purely data-based information on very lowly correlated and very highly correlated metabolite pairs in order to further constrain Eqn. (6).

### Genetic Algorithm

Eqn. (6) is overdetermined since, in reality, the number of zeros in the Jacobian vector is considerably higher than the degrees of freedom of the system. Therefore, an optimization based on the minimization of the Euclidean norm of the difference between left-hand-side and right-hand-side of Eqn. (6) is to be performed. One other proper objective function to calculate Jacobian vector, **j**, from the equation is already-reported sparsity of cellular networks [5], [7], [22]. To this aim, we used a second objective function simultaneously to determine **j**: the maximization of the number of zeros in the unknown vector. We employed Genetic Algorithm for this purpose. Mathematically speaking, our multi-objective function to be optimized is:
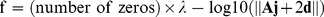
(7)


Note that the two terms in the objective (fitness) function are indeed summed up since the logarithm of the term in parenthesis is negative for values smaller than 1. λ was chosen 0.05 in all simulations. To guarantee the search of reasonable solution space, first term of Eqn. (7) was constrained to have a maximum value of (n^2^−n)×0.9, since the diagonals of a Jacobian matrix cannot be zero, and it is not feasible for a Jacobian matrix to have more than 90% of its remaining entries to be zero. Similarly, the second term was replaced by (10–[10+log10(||**Aj**+2**d**||)]/50) if the residual norm of the individual in question is smaller than 1×10^−10^. Thereby, we reduced the contribution of the second term on the objective function for such small residual norms to shift the emphasis on the number of zeros. These constraints prevented the genetic algorithm to get stuck in local minima. For noise analysis and missing-data analysis cases, we reduced the contribution of residual norms smaller than 1×10^−5^ to the fitness function and used (5–[5+log10(||**Aj**+2**d**||)]/50) as the second term of the objective function defined in Eqn. (7). Since noise or missing data would increase the minimal residual norm that can be achieved, our rearrangement was done to balance this fact.

*ga* function in MATLAB’s Global Optimization Toolbox was used to code the problem in genetic algorithm. A bit-string representation of individuals was used. *ga* was run with 4 different subpopulations simultaneously, each having 50 individuals. Mutation rate was chosen as around 1/(individual length) [23]. A parallelized version of *ga* was run with the help of MATLAB’s Parallel Computing Toolbox. The other parameters of genetic algorithm were used in default, and observed to not to affect the results. Since no interaction means a symmetric entry of two zeros in Jacobian matrix, zeros in the binary individuals were generated symmetrically. The zero-elements of an individual were used as a constraint on Eqn. (6), and MATLAB’s *lsqlin* from its Optimization Toolbox was used to calculate a corresponding candidate Jacobian vector, and the second term in the fitness function. Simulations were performed on a 4-core desktop computer in Windows environment. Convergence was achieved quickly in 100 to 800 generations depending on the size of the network studied. The algorithm of the approach is given in Supporting Information S1.

### Statistical Analysis of Results

The Jacobian vector corresponding to the best individual obtained from the genetic algorithm was compared with the real Jacobian vector, and prediction was quantified statistically by using true positive rate (TPR) and false positive rate (FPR). When necessary, g-score [24] was calculated based on the following formula to allow better comparison:
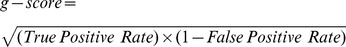
(8)


The directionality was taken into account while calculating these metrics. That is, a true positive count meant that both the availability of interaction and its direction were correctly inferred. Similarly, false negatives were the edges which were either predicted as no-edge, or predicted in wrong directions. The entries in the calculated Jacobian vector which are smaller than 1×10^−8^ were assumed to be zero.

## Results and Discussion

### Demonstration of the Approach on a Small Cellular Network

**Figure 1:**
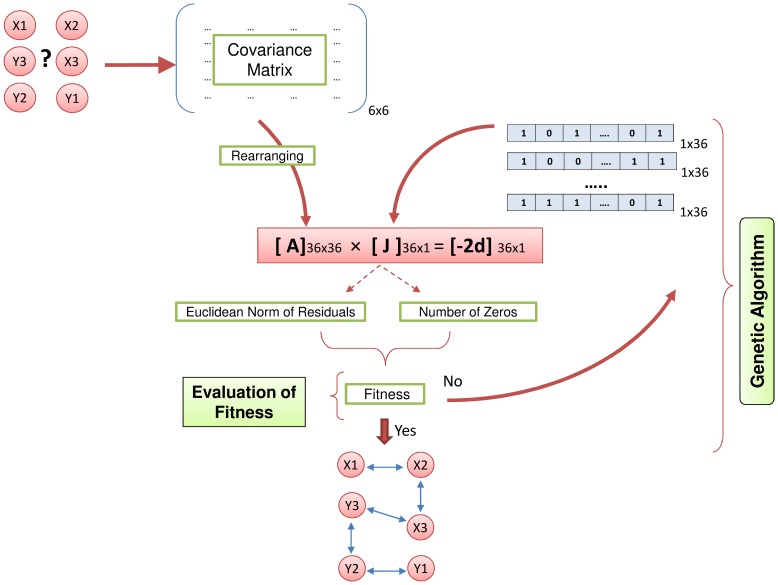
Illustrating Lyapunov-equation based approach to use sparsity as cellular objective to predict underlying network structure. The gene network is from [24]. The genetic-algorithm-coded approach uses covariance matrix as an input to predict interaction strengths (Jacobian matrix) based on a mathematical dual objective of maximal number of zeros and minimal Euclidean norm of the residuals. See also the algorithm presented in Supporting Information S1.

We start with a demonstration of our approach on a smaller (6-node) system. We preferred a non-metabolic system for the demonstration on purpose to draw attention to the fact that our approach can also be applied to the inference of other biological networks such as gene-regulatory or signaling networks. The system has 6 nodes, and 5 interactions in between [25], and was obtained from BioModels database [26]. So, matrix A in Eqn. (6) has dimensions of 36×36, and Jacobian vector to be estimated is a 36×1 vector. The real Jacobian of the system, calculated numerically from the available kinetic model, has 20 zeros, and the degrees of freedom of matrix A is 15. Our approach and its results are also visually demonstrated in Figure 1 via this example system. As detailed in Figure 1, solving modified Lyapunov equation, Eqn. (6), with the double objective function defined in Eqn. (7) using the genetic algorithm has led to the exact Jacobian, meaning a full inference of the directed network structure. Our approach found the solution very fast in only 1 or 2 generations. GGM values of metabolite pairs suggested a link between two pairs, which was used as an input to our algorithm by always keeping the corresponding entries in the individuals 1 for these pairs. When even no such information is used, the exact solution is found in about 10 generations.

### Discovery of three Metabolic Networks from Different Kingdoms

We first calculated covariance matrices of the three metabolic networks *in silico* as mentioned in the Methods section: 12-metabolite brain glycolysis [27], 13-metabolite yeast glycolysis [28], and 18-metabolite *E.coli* central metabolism [29]. Then, we calculated the corresponding full-order partial Pearson correlation matrices based on a simple GGM formulation [30]. The strength of similarity based approaches was used, in a very stringent way, as an input to our algorithm. The use of our stringent cut-offs identified 3 linked and 8 non-linked metabolite pairs for brain, 3 linked and 10 non-linked pairs for yeast, and 5 linked and 41 non-linked pairs for *E. coli*. This information was used as an additional constraint on the standardized Lyapunov Equation (Eqn. (6)) as detailed in Methods section. Table 1 reports true positive rates and false positive rates of the inferred directed networks for these metabolic systems based on the sparsity objective as well as Spearman correlations between the directed interaction strengths. For brain and *S. cerevisiae*, our algorithm led to the exact inference of the network structure, with exact values of interaction strengths (eg. inference of the exact Jacobian matrix) as obvious from the perfect Spearman correlation. For *E. coli*, results led to a very high true positive rate (0.85) with no false positives at all. That is, 33 of 39 real interactions were able to be recovered by our approach. A closer inspection and comparison of real Jacobian matrix and the inferred Jacobian matrix revealed that 5 of the 6 false negatives were due to the wrong assignments of reversibility to irreversible reactions. However, the interaction strengths of the reversible parts of these reactions were very low (on the order of 10^−4^), making these inferred interactions practically irreversible, and in the direction of true edges. This corresponds to a practical TPR of 0.97. The remaining false negative was indeed for a very weak regulatory interaction between fructose-1,6-biphosphate and pyruvate (on the order of 10^−5^). Our approach could not capture this interaction due to its almost-zero strength. This means that our approach is almost flawless for the inference of stronger interactions, considering these three systems from mammalian, eukaryotic and prokaryotic organisms. One should note that the predicted networks are condition-specific. The approach infers the active links of the metabolic networks for the condition of interest rather than inferring the general metabolic network with all possible reactions, which also has a sparse structure.

**Table 1:** Inference results for three metabolic systems.

	System Characteristics		Inference-Quality Metrics		
	Number ofNodes	Number ofInteractions	True Positive Rate	False PositiveRate	Spearman Correlationof Strengths
Brain	12	17	1.00	0	1.00
*S. cerevisiae*	13	21	1.00	0	1.00
*E. coli*	18	39	0.85	0	1.00

Our approach generates networks with very high TPR and no FPRs. Also, there is full correlation between the interaction strengths of real networks and inferred networks.

To allow a clearer demonstration of the positive effect of the sparsity objective on the results, we repeated calculations with an alternative double-objective function which simultaneously minimizes (i) sum of the absolute values of the elements of Jacobian matrix and (ii) Euclidean norm of the residuals, by using a similar framework as in Eqn. (7). Results are associated with very high false positive rates (on the order of 0.30), indicating the clear contribution of sparsity objective on getting promising predictions (results not shown). Additionally, we tested the effect of sparsity term of the fitness function on the results by removing the term from the function and running our algorithm. We have obtained networks with noticeably denser structures. The most obvious characteristics of these predicted networks is that they are associated with very high false positives (a natural result of having denser structure).

A previous study used two types of *in silico* data around steady state generated from the same *E. coli* and *S. cerevisiae* models to infer undirected networks [6]. They reported GGM (n^th^ order partial correlation) as most powerful similarity-based approach based on their analysis. We compared our results with the networks inferred in that study based on GGM. Additionally, we generated *in silico* steady-state data for brain metabolic model, and analyzed the data with GGM approach. Results are compared in Table 2. GGM-based approach results in undirected networks with acceptable TPRs and FPRs. Our directed network inference approach, on the other hand, leads to very promising results using similar steady-state based covariance as input, with almost exact inference of networks including the quantified strength of interactions. (see Table 2). Here, one should keep in mind that in our approach exact covariance matrices were used rather than *in silico* data-based ones. Therefore, our main focus in such a comparison is to demonstrate sparsity as a valid cellular objective function for network inference.

**Table 2:** Comparison of performance of our approach with similarity-based GGM method.

	Lyapunov-based Approach		Similarity-based GGM Approach			
			Enzymatic*		Intrinsic*	
	TPR(directed)	FPR	TPR(directed)	FPR	TPR(directed)	FPR
Brain	1	0	0.64	0.34	0.44	0.03
*S. cerevisiae*	1	0	0.69	0.19	0.77	0.13
*E. coli*	0.85	0	0.66	0.16	0.61	0.08

Note that reported TPRs and FPRs are for directed network inference in our case whereas they are for undirected network inference for GGM method.

In [6], two types of in silico steady-state data were generated. For details, check the related reference.

### Noise Analysis

Next, the sensitivity of our approach to noise in data was analyzed. To do so, we focused on one of the networks: *S. cerevisiae* glycolysis. For the noise analysis, we followed the approach adopted in [31], who added a noise to data from normal distribution with a variance corresponding to a certain percentage of (eg. 50%) the variance of each variable in the data. They then tested the effect of noise on the performance of their GGM approach. Others applied similar noises to metabolomic datasets to test their methods [32], [33]. In our study, the noise analysis was applied to both our Lyapunov-equation based approach, and to similarity-based approaches comparatively. First, we added 50% noise to the two types of *in silico* data reported in [6], and calculated corresponding TPR’s and FPR’s of resulting similarity-based inferred undirected networks. This was repeated 10 times, and the arithmetic averages were calculated, as reported in Table 3. Next, we checked the effect of this noise on covariance matrices of these data. We have observed that such noise causes a normally distributed noise on the independent entries of covariance matrices with mean 1 and standard deviation around 0.005. Therefore, we multiplied our Lyapunov-derived covariance matrix entries for *S. cerevisiae* with random numbers from normal distribution with these properties to mimic a similar noise effect. The resulting covariance matrix was used as the input to our genetic algorithm. After repeating this analysis with 10 different such covariance matrices, resulting TPR and FPR values are averaged and reported in Table 3. Again we remind that, the reported TPRs and FPRs for our Lyapunov-based approach are based on directed-network inference unlike the GGM approach of [6] used for comparison.

**Table 3:** Effect of noise on the inference of *S. cerevisiae* network for our directed approach and for undirected similarity-based GGM approach.

Lyapunov-based Approach(0.5% standard dev.)			Similarity-based GGM Approach					
			Enzymatic Variation			Intrinsic Variation		
TPR	FPR	R_Sp_	TPR	FPR	R_Sp_	TPR	FPR	R_Sp_
0.73*	0.11	0.51+	0.60	0.15	0.39	0.71	0.21	0.47

Results are average of 10 noise-incorporated repetitions. Note that reported TPRs, FPRs and Spearman Correlations (R_sp_) are for directed network inference in our case whereas they are for undirected network inference for GGM method.

Value becomes 0.76 when interaction direction is not considered.

Value becomes 0.69 when interaction direction is not considered.

As expected, and observed before [31], noise has an effect on the performance of network inference approaches. A comparison of the approaches shows that Lyapunov-equation based approach presented in this work has lowest FPR value for the noise-incorporated cases. TPR values may not seem very different at first sight, however, unlike edges correctly inferred by GGM, all the edges correctly inferred by our approach have correct directionality, which makes the higher performance of our approach clear even for the noisy data input. When we calculated TPR of the network inferred by our approach by ignoring directionality, we calculated a value of 0.76, and FPR remained the same. We went further and wanted to see how our approach would behave if even a larger noise with doubled standard deviation is considered. Multiplying covariance matrix entries with random numbers from normal distribution with mean 1 and standard deviation 0.01 resulted in a directed network with TPR of 0.65, and FPR of 0.21, still comparable to the less noisy similarity-based counterparts reported in Table 3.

### Effect of Missing Data

One important and relatively untouched issue in the literature is how network inference approaches behave in case of missing data. It may not be possible to have metabolomic measurement for every node in a metabolic network. We have investigated the effect of missing data on the prediction capacity of our approach, compared to similarity-based approaches.

We have focused on *S. cerevisiae* network, and assumed that no information is available about two nodes in the network: Fructose biphosphate (F16bP), and 2-phosphoglycerate (2-PG). So, we have discarded corresponding columns and rows from the covariance matrix before feeding it to our genetic-algorithm-based approach. F16bP is connected to Fructose-6-phosphate and Phosphate on one side and to Triose-phosphate on the other side in the original network. With the missing data, we expect a link between Fructose-6-phosphate and Triose-phosphate, as well as a link between phosphate and triose-phosphate. 2-PG is connected to 3-phosphoglycerate on one side and to phosphoenolpyruvate on the other side in the original network, so we expect a direct link between these two metabolites in the missing-data case.

The inferred network included a link between 3-phosphoglycerate and phosphoenolpyruvate, and between Fructose-6-phosphate and Triose-phosphate as expected. The expected connection between triose-phosphate and phosphate was not recovered. This is probably due to the strength of the interaction between F16bP and phosphate in the original network: it was a relatively weak interaction. The inferred directed network has a TPR of 0.67 and an FPR of 0.08. When the prediction of directionality is not taken into account, TPR and FPR are 0.74 and 0.08 respectively. This corresponds to a g-score of 0.82. For comparison, *in silico* data from [6] were also analyzed with GGM-based inference in the case of missing information for the F16P and 2-PG nodes. Of the two types of *in silico* data reported there, the enzymatic-data resulted in a TPR of 0.69, and an FPR of 0.31. This corresponds to a g-score of 0.69. GGM achieved the same TPR as the original case (Table 3), but with an almost doubled FPR. For the other data type (termed intrinsic data), TPR was calculated as 0.82, and FPR was calculated as 0.23, corresponding to a g-score of 0.79. Again, a slight change in TPR was associated with a high relative increase in FPR in the case of missing data for the two nodes. Our approach here highly outperformed GGM-based approach in terms of resulting FPRs. Also Spearman correlation between the strengths of the predicted and calculated Jacobian values was 0.56 (p-value: 3×10^−11^) when interaction directions were considered, and 0.70 when interaction directions were not considered (p-value: 2×10^−9^) by our approach. Spearman values of 0.32 and 0.44 by the similarity-based approaches were identified between undirected Jacobian strengths and GGM values.

## Conclusions

We have presented a theoretical analysis which justifies the use of sparsity as a cellular objective from the perspective of network inference. Additionally, the results imply a superiority of our approach to the similarity-based approaches reported for metabolic network inference so far. The approach has three strengths: (i) high-quality directed network inference, (ii) no requirement for advanced and complicated experimental design such as knock-outs, only data around steady-state are sufficient, (iii) recovering interaction strengths between metabolite pairs. Moreover, the approach can readily be applied to the inference of other types of biological networks such as gene-regulatory networks.

One should note that our Lyapunov-based approach has an equation system with n^2^ unknowns for an n-metabolite system. It may seem to be an obstacle to apply the method to metabolomic datasets with larger coverage. However, considering increased computational capacity with novel approaches such as cloud-computing, this may not be a primary issue. Besides, we have shown that our approach can still have a high-TPR & low-FPR characteristics in the case of missing information for some nodes. In this sense, our study has attempted to address the untouched issue of missing data in the metabolic network inference area by covering also the performance of similarity-based approaches on this issue. Our next focus will be the improvement of the algorithm to show its applicability to larger metabolic networks. A further challenge will be to test the approach in terms of the required data characteristics, such as the number of replicates, to infer structures from real metabolome data.

## Supporting Information

Information S1 **Derivation of Modified Lyapunov Equation (** **Eqn. 6** **) from** **Eqn. 5** **, and the algorithm of the developed method.**
(PDF)
