# Supplementary material for: Sparsity as Cellular Objective to Infer Directed Metabolic Networks from Steady-State Metabolome Data: A Theoretical Analysis
Source: PLoS One. 2013 Dec 31;8(12):e84505. doi: 10.1371/journal.pone.0084505 (PMC3877278; doi:10.1371/journal.pone.0084505)
Supplement: Information S1 — Derivation of Modified Lyapunov Equation ( Eqn. 6 ) from Eqn. 5 , and the algorithm of the developed method. (PDF) [file pone.0084505.s001.pdf]

**SUPPORTING INFORMATION S1**

**1. Derivation of Modified Lyapunov Equation: Equation (6)**

$$\mathrm{vec}(\mathbf{J}\Gamma + \Gamma\mathbf{J}^T = -2\mathbf{D})$$

$$\mathrm{vec}(\mathbf{J}\Gamma + \Gamma\mathbf{J}^T) = \mathrm{vec}(-2\mathbf{D})$$

$$\mathrm{vec}(\mathbf{J}\Gamma) + \mathrm{vec}(\Gamma\mathbf{J}^T) = -2\mathbf{d}$$

with **d** being vectorized form of **D**.

Rule 1: $\mathrm{vec}(\mathbf{ABC}) = (\mathbf{C}^T \otimes \mathbf{A})\ \mathrm{vec}(\mathbf{B})$

Rule 2: Multiplication with identity matrix will not affect the equality.

Applying those rules;

$$\mathrm{vec}(\mathbf{IJ\Gamma}) = (\mathbf{\Gamma}^T \otimes \mathbf{I})\,\mathrm{vec}(\mathbf{J})$$

$$\mathrm{vec}(\mathbf{\Gamma J}^T\mathbf{I}) = (\mathbf{I} \otimes \mathbf{\Gamma})\,\mathrm{vec}(\mathbf{J}^T)$$

$$(\mathbf{\Gamma}^T \otimes \mathbf{I})\,\mathrm{vec}(\mathbf{J}) + (\mathbf{I} \otimes \mathbf{\Gamma})\,\mathrm{vec}(\mathbf{J}^T) = -2\mathbf{d}$$

Rule 3: $\mathrm{vec}(\mathbf{J}) = \mathbf{P}\,\mathrm{vec}(\mathbf{J}^T)$ with **P** being a permutation matrix

$$(\mathbf{\Gamma}^T \otimes \mathbf{I})\,\mathrm{vec}(\mathbf{J}) + (\mathbf{I} \otimes \mathbf{\Gamma})\,\mathbf{P}\,\mathrm{vec}(\mathbf{J}) = -2\mathbf{d}$$

Hence,

$\mathbf{A} = (\mathbf{\Gamma}^T \otimes \mathbf{I}) + (\mathbf{I} \otimes \mathbf{\Gamma})\mathbf{P} \;=\; (\mathbf{\Gamma} \otimes \mathbf{I}) + (\mathbf{I} \otimes \mathbf{\Gamma})\mathbf{P}$ (since **Γ** is symmetric, its transpose is the same)

For a 2 x 2 system, given that covariance matrix, **Γ**, is of the form $\begin{bmatrix} a & b \\ b & d \end{bmatrix}$, eqn.(5) with Jacobian matrix in vectorized form looks as follows;

$$\begin{bmatrix} a & b & 0 & 0 \\ b & d & 0 & 0 \\ 0 & 0 & a & b \\ 0 & 0 & b & d \end{bmatrix}\begin{bmatrix} j_{11} \\ j_{12} \\ j_{21} \\ j_{22} \end{bmatrix} + \begin{bmatrix} a & b & 0 & 0 \\ 0 & 0 & a & b \\ b & d & 0 & 0 \\ 0 & 0 & b & d \end{bmatrix}\begin{bmatrix} j_{11} \\ j_{12} \\ j_{21} \\ j_{22} \end{bmatrix} = -2\begin{pmatrix} 1 \\ 0 \\ 1 \\ 0 \end{pmatrix}$$

The addition of two coefficient matrices appearing in the equation above gives **A** matrix of eqn. (6).

## 2. The Algorithm of the Developed Method to Infer the Network based on Lyapunov Equation

**Input:** covariance matrix, fluctuation matrix

**Output:** Jacobian vector (j)

**Algorithm:**

1. Fix the length of the individuals of Genetic Algorithm (GA), to be represented as bit-string.
2. Convert the input covariance matrix to GGM (Graphical Gaussian Model) correlation matrix
3. Check GGM-type correlations with >0.60 and < 0.001

   IF >0.60

   Make the corresponding symmetric entries in binary individuals 1

   ELSEIF < 0.001

   Make the corresponding symmetric entries in binary individuals 0.
4. Determine the number of entries of the individuals that was not assigned a value. This will be the input parameter to GA as the length of individuals.
5. Enter parameters for GA:

   Population Size (150), Number of Generations (800), Mutation Rate (1/IndividualLength), Elite Count (3), Individual Length, Other Parameters: default
6. Do GA to assign binary values to the rest of entries in the original individual vectors
7. For each generated individual,
   a. Apply least square solution to Aj-d by fixing the entries of j which corresponds to zeros of the individual vector to zero.
   b. Calculate the values of each of the two terms of the fitness equation (Eqn.7) for the individual.

c. Replace the first term by $(n^2-n)x0.9$ if it is greater than this value.

d. Replace the second term by $(10 – [10 + log10(||Aj+2d||)]/50)$ if the residual norm of the individual is smaller than $1x10^{-10}$.

e. Calculate the fitness of the individual.

8. Repeat 6. and 7. for the entered Number of Generations.
9. Select best individual in the last population, and record the corresponding j.
10. Compare it with the true Jacobian vector, calculate TPR, FPR.
